# Electromyography as a Means of Assessing Masticatory Muscle Activity in Patients with Pain-Related Temporomandibular Disorders

**DOI:** 10.1155/2020/9750915

**Published:** 2020-08-13

**Authors:** Liliana Szyszka-Sommerfeld, Monika Machoy, Mariusz Lipski, Krzysztof Woźniak

**Affiliations:** ^1^Department of Orthodontics, Pomeranian Medical University of Szczecin, Al. Powstańców Wlkp. 72, 70111 Szczecin, Poland; ^2^Department of Preclinical Conservative Dentistry and Preclinical Endodontics, Pomeranian Medical University of Szczecin, Al. Powstańców Wlkp. 72, 70111 Szczecin, Poland

## Abstract

**Aim:**

The aim of this study was to evaluate masticatory muscle electrical activity in patients with pain-related and pain-free temporomandibular disorders (TMDs) as well as in subjects with no TMD.

**Methods:**

Ninety children with mixed dentition were recruited to the study. Of this total, 30 subjects were diagnosed with pain-related TMD (TMD-P), 30 with pain-free TMD (TMD-PF), and 30 without TMD. We used Axis I of the Research Diagnostic Criteria for TMD (RDC/TMD) to assess the presence of TMD in the examined children. The electromyographical (EMG) potentials of the temporalis and masseter muscles were measured with a DAB-Bluetooth Instrument (Zebris Medical GmbH, Germany) at rest and during maximum voluntary clenching (MVC).

**Results:**

An analysis of the EMG recordings showed statistically significant intergroup differences in masticatory muscle electrical activity at rest and during MVC. Significantly higher rest temporalis muscle activity was noted in pain-related TMD subjects compared with that children from the pain-free TMD and non-TMD groups, as well as in TMD-PF children in relation to those without TMD. The EMG potentials of the temporalis muscle during MVC were much lower in patients with TMD-P than in pain-free TMD and non-TMD subjects. Masseter muscle activity at rest in the TMD-pain group was significantly greater, and masseter muscle EMG potentials during clenching were markedly lower than in patients with no TMD diagnosis.

**Conclusion:**

The use of electromyography to assess masticatory muscle function revealed alterations in the pattern of temporalis and masseter muscle activity in patients with pain-related TMD compared with the pain-free subjects.

## 1. Introduction

Temporomandibular disorders (TMDs) are associated with a number of clinical conditions that affect the stomatognathic system, in particular the masticatory muscles and the temporomandibular joint (TMJ) as well as associated structures [1–3]. The principal signs and symptoms of TMDs are muscle and joint tenderness or pain, joint noises, and disturbances in mandibular movements. The pain associated with TMD is persistent, recurring, or chronic in nature and not only concerns the TMJ and masticatory muscles but may also radiate to adjacent structures such as the teeth, ears, the neck, temples, forehead, and back muscles [2–4].

Factors that may play an important role in TMD multifactorial aetiology include traumas, local conditions such as occlusal interferences as well as systemic, iatrogenic, and psychological aspects [5–7].

Temporomandibular disorders are the main nondental cause of orofacial pain in children and adolescents [8]. The prevalence of TMD-signs and symptoms is rare in early childhood but becomes more in adolescence and adulthood. Previous epidemiological studies have reported subjective symptoms of TMD in between 1% and 50% of children and adolescents in the general population and pain-related TMD in between 1% and 22% of the youngest group of subjects [9–14]. The prevalence of TMD-signs in children and adolescents ranges from 3% to 33% [10–12, 15].

One of the most advanced and useful diagnostic tools providing clinical and research criteria for objective TMD assessment are the Research Diagnostic Criteria for Temporomandibular Disorders (RDC/TMD) [2, 16]. This tool helps identify both physical and psychosocial aspects, standardizes the procedures followed in epidemiological studies, and has shown acceptable reliability in children and adolescents [17, 18]. Surface electromyography (sEMG) is widely applied as an additional noninvasive tool for assessing patients with TMD as well as for observing the electrophysiological behaviour of muscles under a variety of physiological conditions [19–25]. Due to the simplicity of this method and its safety and availability, it has also been used in studies on children [26–28]. An EMG evaluation of masticatory muscle function in TMD subjects provides a basis for diagnosing the disease, monitoring its progression and measuring the effectiveness of treatment. Numerous studies have shown that patients with TMD exhibit alterations in masticatory muscle EMG activity either as a result of the disorder itself or due to a compensatory mechanism associated with the symptoms [20, 29–32]. It has been demonstrated that individuals with pain-related TMD may alter the recruitment of their masticatory muscles as a result of sensorial-motor interactions, the pain associated with which it can modify the formation of action potentials and, possibly, myoelectric activity [33, 34]. In this context, it is important to determine the masticatory muscle electrical activity in patients with TMD, including pain-related TMD and pain-free TMD subjects.

To the authors' knowledge, only a few studies have been conducted on masticatory muscle function in the children with TMD [26, 27, 35]. As a consequence, research on the electromyographical activity of the masticatory muscles in such subjects with TMD problems is needed. Investigating the electromyographic features of children with TMD is key to early identification of problems that predispose such patients to pain and muscle/joint dysfunction in adulthood as well as to the development of treatment strategies that can improve their muscle function and prevent persistent TMD later in life [26, 35].

The aim of the study was to evaluate the EMG activity of the masticatory muscles in children with pain-related and pain-free TMD as well as in subjects with no TMD. We hypothesized that, in the case of EMG potentials of the temporalis and masseter muscles at rest and during maximum clenching, no differences exist between the analysed groups of patients.

## 2. Materials and Methods

### 2.1. Ethical Approval

The study protocol was approved by the Local Bioethics Committee of the Pomeranian Medical University and was assigned the number KB-0012/08/15. All the children's parents gave their informed consent to all the procedures performed. This clinical research was also registered as a case-control study in the ClinicalTrials.gov database and was assigned the number NCT04409067.

### 2.2. Participants

Ninety children with mixed dentition were recruited to the study. The subjects had been referred to the Orthodontic Clinic in Szczecin, Poland, for orthodontic treatment. We used Axis I of the Research Diagnostic Criteria for TMD (RDC/TMD) to assess the presence of TMD in the examined children. All the subjects were subdivided into three nonoverlapping groups: a TMD-pain group, a pain-free TMD group, and a non-TMD group. The groups were matched for age and gender. The inclusion criteria for all groups were mixed dentition (the subjects should be aged between 7 and 12 years) and express consent to participate voluntarily in the study. The TMD-pain group consisted of 30 children (16 girls and 14 boys) aged between 7.1 and 12.3 (mean 8.8 ± 1.5) with a pain-related TMD diagnosis. All the patients in the TMD-pain group had myogenous or arthrogenous TMD according to the RDC/TMD protocol. The pain-free TMD group consisted of 30 children (14 girls and 16 boys) between 7.3 and 12.6 years of age (mean 9.0 ± 1.3). To be included in the pain-free TMD group, the participants had to meet Axis I of the RDC/TMD criteria for a pain-free TMD diagnosis. The non-TMD group comprised 30 children (15 girls and 15 boys) aged between 7.2 and 12.5 (mean 8.9 ± 1.6) without any recognised TMD based on RDC/TMD, Axis I. Excluded from these study groups were subjects who had undergone orthodontic or masticatory motor system dysfunction treatment, systemic or rheumatologic diseases, a history of mouth breathing, surgery, traumas, or malformations in the head and neck regions.

### 2.3. Clinical and EMG Examination

The function of the stomatognathic system was assessed by means of a clinical and electromyographic examination. In the first part of the clinical examination, we took the general medical history of the patients. This included information on subjective TMD symptoms are jaw pain during functional activities, occurrence of frequent headaches, stiffness/fatigue of the jaw, limited mouth opening, grinding or clenching of teeth, and possible presence of TMJ noises. During the second part of the clinical examination, the children were diagnosed with one or more disorders according to RDC/TMD Axis I: Group I: muscle disorders (Ia with myofascial pain; Ib with myofascial pain with limited opening), Group II: disc displacements (IIa with reduction; IIb without reduction with limited opening; IIc without reduction but without limited opening), and Group III: other common joint disorders (IIIa arthralgia; IIIb/IIIc arthritis) [2, 17]. All the children were examined by a single trained assessor. The clinical examination performed using the Axis I RDC/TMD protocol included an assessment of pain on palpation, a measurement of mandibular, the range of motion, an evaluation of pain and joint noises during mandibular movements, and tenderness induced by muscle and TMJ palpation. Finally, based on the self-report, clinical criteria, and diagnosis, the pain-related TMD group included children with myalgia Ia, Ib (pain of muscular origin, including pain experienced in the masticatory muscles or face at rest and during functional activities, as well as pain associated with localized areas that are tender to palpation in the muscle at 3 or more sites), as well as arthralgia IIIa (joint pain during palpation and joint-related pain during mouth opening or during lateral excursion). The pain-free TMD group comprised children diagnosed with disc displacements IIa, IIb, and IIc.

Replicate assessments of clinical signs of TMD were recorded for 20 randomly selected patients in order to assess intraexaminer reliability. For this purpose, intraclass correlation coefficients (ICCs) were calculated for both continuous and dichotomous variables of the RDC/TMD examination. According to the guidelines for interpretation for ICC values, reliability was classified as “poor” (ICC < 0.4), “fair to good reliability” (0.4 ≤ ICC ≤ 0.75), or “excellent” (ICC > 0.75) [36, 37].

An intraoral examination was performed to assess occlusal characteristics in all the participants, including Angle's classification, posterior crossbite, overbite, overjet, and lateral open bite.

In the next part of the examination, electromyographic recordings with a DAB-Bluetooth Instrument (Zebris Medical GmbH, Germany) were taken for all the study subjects by a single experienced examiner. The protocol followed here has already been described by us in previous papers [27, 28, 38].

The EMG assessment was recorded with a bipolar surface electrodes (silver/silver chloride - Ag/AgCl, disposable, self-adhesive electrodes with a fixed interelectrode distance of 20 mm, a Noraxon Dual Electrode, Noraxon, USA) bilaterally placed on the children's skin above the body of the masseter muscle and the anterior temporalis muscle, running parallel to muscle fibers. According to Ferrario et al. [39], in the case of the temporalis anterior muscle, the electrodes were located vertically on both sides along the anterior margin of the muscle, while for the masseter muscle, the electrodes were placed paralell to the muscle fibers with the upper pole of the electrode located at the intersection between the tragus-labial commissura and exocanthion-gonion lines. A reference electrode was situated inferior and posterior to the right ear.

The skin of the patients was cleaned with 70% ethyl alcohol prior to the placement of the surface electrodes, and then an impedance test was performed with a Metex P-10 measuring device (Metex Instruments Corporation, Korea). Five minutes later, the EMG recordings commenced. The EMG measurements of the temporalis and masseter muscles were performed at rest and during maximum voluntary clenching (MVC) in the intercuspal position and on cotton rolls. For both recordings during MVC, the subjects were invited to clench as hard as possible for 5 seconds. The recording during MVC on the cotton rolls was used for normalization purposes. To standardize the EMG potentials of the masticatory muscles with tooth contact, two 10 mm thick cotton rolls were placed on the mandibular second premolars and molars or on the mandibular second milk molars and the first permanent molars of each participant, and 5 seconds of maximum clenching was recorded. Finally, the mean values of the EMG potentials (raw data) of the temporalis and masseter muscles measured both at rest and during MVC which were expressed as a percentage of the mean potentials (reference values) measured during the standardization test (clenching on the cotton rolls) according to the following formula: mean values (*μ*V) at rest or during MVC/mean values (*μ*V) during MVC on two cotton rolls *x* 100% (unit *μ*V/*μ*V%) [39].

A rest period of at least 5 minutes was allowed between each recording. The EMG recordings were repeated for all children at least three times. The EMG values obtained during the last two EMG measurements were averaged.

The DAB-Bluetooth Instrument was hooked up to a computer to process the data and present them graphically. The EMG signal was amplified, digitized, and digitally filtered.

The asymmetry between the activity of the left and right masticatory muscles was quantified by the Asymmetry Index (As, unit %, range from 0% to 100%), according to the following equation: As=∑_*i*=1_^*N*^|*R*_*i*_ − *L*_*i*_|/∑_*i*=1_^*N*^(*R*_*i*_+*L*_*i*_) × 100 [40].

The repeatability of the recording measurements was tested by ensuring that the same examiner performed duplicate EMG evaluations on the 20 subjects. The two EMG measurements were separated by a gap of 15 minutes. The data obtained from the repeated evaluations were presented as the normalized mean values of masticatory muscle activity at rest and during MVC. The repeatability of electrode localization was maintained by applying a standard scheme for the positioning of the surface electrodes [41].

### 2.4. Statistical Analysis

The Kolmogorov–Smirnov test was applied to determine the normality of data distribution. After determining the normality of the distributions, the mean values and standard deviations (SD) in *μ*V/*μ*V% for the normalized EMG values were compared and analysed. When comparing a single pair of mean values, the Student's *t* test was applied. Analysis of variance (ANOVA) was used when multiple comparisons were intended. When ANOVA indicated a significant difference, a StudentNewmanKeuls post-hoc test was performed, with the level of significance set at 5% (*P*=0.05). The differences in the prevalence of occlusal characteristics between the groups were determined by means of the chi-squared test.

## 3. Results

The reliability value for the RDC/TMD clinical examination ranged from good to excellent (from 0.68 to 1.0).

The results of the intraoral examinations for the groups are presented in Table 1. No significant intergroup differences were observed with regard to the prevalence of occlusal characteristics (*P* > 0.05).

Table 2 presents data on the repeatability of the recorded EMG measurements. No differences were noted between the two repeated EMG recordings when it came to masticatory muscle activity at rest and during MVC (*P* > 0.05).

The normalized EMG data, i.e., the activity of the temporalis and masseter muscles at rest and during MVC for the TMD and non-TMD groups, are shown in Tables 3 and 4.

An analysis of the EMG recordings revealed statistically significant intergroup differences in temporalis and masseter muscle EMG activity at rest (for the temporalis muscles *P* ≤ 0.001; for the masseter muscles *P*=0.003). Significantly higher rest temporalis muscle activity was observed in the pain-related TMD patients (7.87 *μ*V/*μ*V%) than in the pain-free TMD group (6.66 *μ*V/*μ*V%; *P*=0.018) and the TMD-free group (5.44 *μ*V/*μ*V%; *P* ≤ 0.001), as well as in TMD-PF children in relation to those without TMD (*P*=0.013). The TMD-pain group exhibited significantly higher masseter muscle EMG potentials at rest (6.03 *μ*V/*μ*V%) compared with the non-TMD group (4.17 *μ*V/*μ*V%; *P* ≤ 0.001) (Table 3).

During MVC, considerable disparities in the EMG potentials of the temporalis and masseter muscles were observed between the analysed groups (for the temporalis muscles *P*=0.003; for the masseter muscles *P*=0.030). Temporalis muscle activity during MVC was significantly lower in children with pain-related TMD (98.49 *μ*V/*μ*V%) in relation to pain-free TMD subjects (114.38 *μ*V/*μ*V%; *P*=0.036) and non-TMD children (129.57 *μ*V/*μ*V%; *P* ≤ 0.001). Furthermore, masseter muscle activity during clenching was much lower in the TMD-pain group (95.42 *μ*V/*μ*V%) than in the TMD-free group (119.43 *μ*V/*μ*V%; *P*=0.006) (Table 4).

There were no significant intergroup differences in the Asymmetry Index for the temporalis and masseter muscles at rest and during MVC (*P* > 0.05) (Tables 3 and 4). Nor were there any major differences between girls and boys in each group with regard to the EMG potentials of the temporalis and masseter muscles in the rest position and during clenching (*P* > 0.05) (Tables 3 and 4).

## 4. Discussion

In the present study, surface electromyography (sEMG) was used to evaluate masticatory muscle activity in children diagnosed with TMD according to the RDC/TMD algorithm. The advantage of global electromyography is its noninvasiveness, because it uses surface electrodes located on the surface of the skin which is absolutely vital in studies involving a cohort of children [19]. We compared 3 groups of patients: pain-related TMD, pain-free TMD, and TMD-free. The results show that it is important to take into account alterations in the electromyographic potentials of the temporalis and masseter muscles in TMD subjects. We demonstrated that masticatory muscle electrical activity varied between the pain and pain-free groups. The EMG activity of the temporalis and masseter muscles at rest in subjects with a TMD-pain diagnosis was higher than in the pain-free groups. This hyperfunction of the masticatory muscles may be associated with a need for greater muscle recruitment in children diagnosed with pain-related TMD in the mandibular rest position [42, 43]. Minimal rest electromyographic activity of the temporalis and masseter muscles observed in children without TMD may indicate a balance between elevator and depressor muscles of the mandible [44]. We also observed that pain-related TMD children had lower masticatory muscle electrical potentials during MVC when compared with the pain-free patients. It was reported in previous studies that the masticatory muscles of symptomatic TMD patients were less efficient and lower EMG activity during clenching may indicate a reduction in their muscle force [45–47]. It was suggested that the bite-force increases in relation to muscle activity [48]. Muscle forces affect the structures of the stomathognatic system and may induce excessive loading on the tooth row and TMJs [49]. In our study, the reduced electrical potentials of the masticatory muscles observed in children diagnosed with pain-related TMD when clenching would suggest that a lower bite-force is to be expected. In this way, the alterations in temporalis and masseter muscle recruitment in the TMD-P subjects during MVC may be considered an effective mechanism of protection for damaged TMJs. Muscle forces are directed to minimize joint loads and muscular efforts as a normal protective control [50].

To date, information regarding masticatory muscle EMG activity in TMD children and adolescents is limited [26, 27, 35]. Early electromyographic analysis of the masticatory muscles in such patients with TMD problems is important for a better understanding of TMD neuromuscular characteristics in this age group and could ensure simpler and improved treatment procedures aimed at addressing muscle involvement in TMD and prevent chronic muscle/joint dysfunction in adulthood [26]. Our study reports on masticatory muscle activity in children with different TMD diagnoses depending on the occurrence of pain based on the Axis I RDC/TMD criteria. As there have been no similar studies, it is difficult to compare our results with others. Moreover, the comparisons are also complicated by the fact that some earlier studies did not include sEMG signal normalization. It is important that a proper EMG assessment should only be carried out with standardized (normalized) values, thereby providing information on the impact of occlusion on neuromuscular activity and ensuring removal most of biological and technical noise, such as anatomical variations, electrode position, nd skin and electrode impedance [51]. The normalization process is necessary for the preliminary processing of raw values to ensure intercomparisons and further analysis. In our study, to standardize the EMG potentials of the masticatory muscles with tooth contact, the subjects were asked to clench on two cotton rolls positioned on mandibular molars [39]. Normalization involved relating the electrical potentials of the muscles to the reference values obtained from the EMG measurements detected during the standardization recordings, that is, MVC with a control substance (cotton rolls). It has been reported that the EMG potentials collected in MVC have the best repeatability. Among the different protocols, an maximum voluntary clenching on cotton rolls has been reported to have the lowest interindividual variability, and for that reason, this method is now commonly used [24, 39, 51–53]. Nevertheless, our findings could be referred to those of Chaves et al. [26], who investigated differences in the electrical activity of the temporalis, masseter, and suprahyoid muscles in both children diagnosed with TMD based on the Axis I RDC/TMD criteria as well as in non-TMD patients. Thirty-four children aged 8–12 years were recruited in the study—17 children with TMD and 17 non-TMD subjects. The EMG raw and normalized data were obtained at rest and during maximum clenching. In contrast to our own study, Chaves et al. found no differences in the EMG values of the masticatory muscles during rest and clenching between patients with TMD and non-TMD subjects. In the case of TMD patients, they observed a lower mean electromyographic ratio for masseter muscles and anterior temporalis muscles (sEMG-M/AT ratio) during MVC. Lauriti et al. [35] evaluated the EMG activity of the masticatory muscles in adolescents with TMD. They recorded masticatory muscle activity in 42 participants aged 14 to 18 years with different degrees of TMD severity based on the Helkimo Index. The authors observed significant intergroup differences in the EMG potentials of the analysed muscles in the rest position and during maximum intercuspation. Their findings suggest that patients with TMD, especially those with more severe symptoms, exhibit masticatory muscle hyperactivity. One study [27] assessed of the temporalis and masseter muscle electrical activity in cleft lip and palate children diagnosed with pain-related TMD according to RDC/TMD criteria. The authors reported that, compared with non-TMD subjects, the EMG activity of the masticatory muscles in TMD-P children was higher at rest while temporalis muscle activity during MVC was lower. Moreover, they observed a significant increase in the Asymmetry Index for masticatory muscle rest activity in the TMD-pain group.

Numerous studies have demonstrated alterations in the EMG potentials of the masticatory muscles of adult patients with pain-related TMD [20, 25, 31, 32, 34, 54–57]. Glaros et al. [54] found that the electrical activity of the left temporalis and left masseter muscles at rest in TMD patients with myofascial pain was significantly higher than in pain-free controls. Similarly, Bodéré et al. [32] observed that the EMG potentials of the temporalis and masseter muscles in the rest position were far higher in adult patients with myofascial or neuropathic pain compared with healthy TMD-free subjects. The authors also found a significant difference in the EMG activity between the pain-free disc derangement disorders group and the pain groups (neuropathic and myofascial) for both muscles except for the masseter muscle of the neuropathic group. Furthermore, they noted significantly higher electrical activity at rest in patients with bilateral pain in relation to subjects with unilateral pain. Berni et al. [31] observed that women with myogenous TMD exhibited significantly greater electrical activity of the temporalis, masseter, and suprahyoid muscles at rest than women without TMD, whereas masseter muscle EMG activity during MVC recorded on parafilm in a TMD group of patients was significantly lower than in non-TMD subjects. Likewise, the results of a study published by Rodrigues et al. [34] revealed higher EMG potentials of the temporalis and masseter muscles at rest in patients with pain-related TMD compared with those of TMD-free subjects. Moreover, no differences were observed between TMD and non-TMD groups in terms of the masticatory muscle EMG activity during MVC. On the other hand, Majewski and Gale [55] reported no significant differences in temporalis rest electrical activity between TMD-pain patients and controls. Manfredini et al. [20] measured the EMG activity of the temporalis and masseter muscles in 36 adult patients diagnosed with myofascial pain based on the RDC/TMD criteria and 36 TMD-free asymptomatic subjects. They also did not observe any disparities in the electrical potentials of masticatory muscles at rest between TMD-pain and non-TMD patients, while EMG activity levels during clenching tasks were significantly greater in subjects with no TMD.

Tartaglia et al. [25] performed EMG recordings of the masseter and temporalis muscles during MVC in 103 patients aged 15–70 subdivided according to RDC/TMD criteria into 3 groups: myogenous, arthrogenous, and psychogenous patients. These groups in turn were compared with 32 control patients aged 19–69 without TMD. The authors found that, during clenching, the masticatory muscles of non-TMD subjects were characterised by much higher normalized EMG potentials and their temporalis muscles had greater symmetry than was the case with TMD patients. Tartaglia et al. suggested that electromyography of the masticatory muscles exhibits its diagnostic usability in an objective discrimination between different RDC/TMD subgroups. In another study by Tartaglia et al. [56], the authors assessed the EMG activity of the temporalis and masseter muscles in 30 patients with a mean age of 23 years diagnosed with arthrogenous TMD and long-term pain as well as in 20 patients aged 19–31 with no signs or symptoms of TMD. They observed that young adult TMD patients exhibited higher and more asymmetric normalized activity of the temporalis muscles during MVC compared with non-TMD controls. Santana-Mora et al. [57] found that the masticatory muscles of non-TMD individuals had higher EMG potentials than was the case with chronic pain individuals with unilateral TMD when clenching. Calculations based on the Asymmetry Index showed that patients with right-sided TMD exhibited preferential use of their left-sided masticatory muscles, whereas patients with left-sided TMD favoured their right-sided temporalis and masseter muscles.

In summary, the findings of the abovementioned reports as well as the results of our study confirmed the existence of differences in masticatory muscle electrical activity between pain-related TMD and pain-free subjects.

The similar intergroup prevalence of occlusal features observed in our report allows us to assume the absence of any relationship between alterations in masticatory muscle activity and malocclusions. However, we did not take into account all malocclusion-related factors. Moreover, another possible limitation of the study may be the fact that the pain-related TMD group included both myogenous and arthrogenous TMD children, since EMG muscle activity may vary in these subgroups of subjects. As a consequence, further studies would be needed to verify our study results.

## 5. Conclusions

An analysis of the EMG recordings revealed significant intergroup disparities in temporalis and masseter muscle electrical potentials at rest and during MVC. Children diagnosed with pain-related TMD exhibited significantly greater EMG activity in temporalis muscles at rest compared with those with the pain-free TMD and non-TMD groups while temporalis muscle electrical potentials when clenching were much lower. Masseter muscle activity at rest in pain-related TMD subjects was significantly higher, and masseter muscle EMG potentials during MVC were markedly lower than in children with no TMD diagnosis.

The use of electromyography to assess masticatory muscle function revealed alterations in the pattern of temporalis and masseter muscle activity in patients with pain-related TMD compared with the pain-free subjects.

## Figures and Tables

**Table 1:** The intraoral examination results for the examined groups of children.

Variable		TMD-pain group		Pain-free TMD group		Non-TMD group	
		*n*	%	*n*	%	*n*	%
Vertical overlap	0–3 mm	12	40.0	14	46.7	16	53.3
	≥3 mm	13	43.3	12	40.0	10	33.3
	Reverse	5	16.7	4	13.3	4	13.3
Overjet	0–3 mm	11	36.7	11	36.7	14	46.7
	≥3 mm	14	46.7	14	46.7	12	40.0
	Negative	5	16.7	5	16.7	4	13.3
Posterior crossbite	No	17	56.7	19	63.3	21	70.0
	Yes	13	43.3	11	36.7	9	30.0
Angle class	I	14	46.7	15	50.0	18	60.0
	II	11	36.7	10	33.3	8	26.7
	III	5	16.7	5	16.7	4	13.3
Lateral open bite	No	26	86.7	26	86.7	27	90.0
	Yes	4	13.3	4	13.3	3	10.0

**Table 2:** The repeatability of the recording measurements.

Region	Activity	1 examination		2 examination		*P* value
		Mean (*μ*V/*μ*V%)	SD	Mean (*μ*V/*μ*V%)	SD	
TA	Rest	6.43	2.29	6.45	2.21	0.978
	MVC	107.30	33.70	107.90	33.60	0.959
MM	Rest	5.32	2.71	5.42	2.66	0.910
	MVC	101.00	27.60	101.90	27.60	0.919

TA: temporalis anterior muscles; MM: masseter muscles; MVC: maximum voluntary contraction.

**Table 3:** Electrical activity of the temporalis and masseter muscles at rest for the examined groups of children.

Region	Variable	Gender	TMD-pain group			Pain-free TMD group			Non-TMD group		
			*n*	Mean	SD	*n*	Mean	SD	*n*	Mean	SD
TA	EA	Girls	16	8.01	1.68	14	6.73	1.76	15	5.54	1.25
		Boys	14	7.71	2.15	16	6.60	2.20	15	5.34	2.07
		Total	30	7.87	1.89	30	6.66	1.97	30	5.44	1.68
	AI	Girls	16	10.86	5.18	14	10.10	6.54	15	11.32	6.66
		Boys	14	14.59	8.10	16	12.20	7.16	15	9.66	4.25
		Total	30	12.60	7.54	30	11.13	6.53	30	10.49	6.36
MM	EA	Girls	16	6.01	2.40	14	5.28	2.25	15	3.90	1.92
		Boys	14	6.05	2.04	16	4.81	1.90	15	4.43	1.90
		Total	30	6.03	2.21	30	5.03	2.08	30	4.17	1.90
	AI	Girls	16	10.10	6.64	14	12.11	6.59	15	15.00	9.67
		Boys	14	10.29	5.89	16	12.84	7.91	15	14.74	8.72
		Total	30	10.19	6.36	30	12.50	7.97	30	14.87	7.52

TA: temporalis anterior muscles; MM: masseter muscles; EA: electrical activity (*μ*V/*μ*V%); AI: asymmetry index (%).

**Table 4:** Electrical activity of the temporalis and masseter muscles during maximum voluntary contraction (MVC) for the examined groups of children.

Region	Variable	Gender	TMD-pain group			Pain-free TMD group			Non-TMD group		
			*n*	Mean	SD	*n*	Mean	SD	*n*	Mean	SD
TA	EA	Girls	16	101.80	29.80	14	113.70	35.50	15	130.00	35.20
		Boys	14	94.70	21.60	16	115.00	27.80	15	129.10	51.20
		Total	30	98.49	31.61	30	114.38	31.09	30	129.57	43.20
	AI	Girls	16	14.74	8.58	14	11.29	5.51	15	16.56	9.83
		Boys	14	7.86	4.99	16	9.59	4.82	15	9.24	5.03
		Total	30	11.53	7.90	30	10.38	5.01	30	12.90	6.80
MM	EA	Girls	16	96.20	28.80	14	110.20	43.70	15	118.50	25.20
		Boys	14	94.50	29.40	16	102.10	32.60	15	120.40	45.90
		Total	30	95.42	28.58	30	105.88	37.71	30	119.43	36.42
	AI	Girls	16	13.85	8.95	14	9.41	5.65	15	9.24	6.83
		Boys	14	9.51	5.49	16	6.10	4.09	15	7.27	4.54
		Total	30	11.82	6.26	30	7.64	4.08	30	8.26	4.59

TA: temporalis anterior muscles; MM: masseter muscles; EA: electrical activity (*μ*V/*μ*V%); AI: asymmetry index (%).

## Data Availability

The datasets supporting the conclusions of this article are included within the article. Access to other data will be considered by the corresponding author upon request.
